# Genomic Analysis of *Pseudomonas asiatica* JP233: An Efficient Phosphate-Solubilizing Bacterium

**DOI:** 10.3390/genes13122290

**Published:** 2022-12-05

**Authors:** Linlin Wang, Fangyuan Zhou, Jianbo Zhou, Paul R. Harvey, Haiyang Yu, Guangzhi Zhang, Xinjian Zhang

**Affiliations:** 1 Shandong Provincial Key Laboratory of Applied Microbiology, Ecology Institute, Qilu University of Technology (Shandong Academy of Sciences), Ji’nan 250103, China; 2 College of Plant Protection, Shanxi Agricultural University, Taiyuan 030031, China; 3 CSIRO Agriculture and Food, Glen Osmond, SA 5064, Australia

**Keywords:** phosphate-solubilizing bacteria, *Pseudomonas asiatica*, genome analysis, plant-growth promotion

## Abstract

The bacterium *Pseudomonas* sp. strain JP233 has been reported to efficiently solubilize sparingly soluble inorganic phosphate, promote plant growth and significantly reduce phosphorus (P) leaching loss from soil. The production of 2-keto gluconic acid (2KGA) by strain JP233 was identified as the main active metabolite responsible for phosphate solubilization. However, the genetic basis of phosphate solubilization and plant-growth promotion remained unclear. As a result, the genome of JP233 was sequenced and analyzed in this study. The JP233 genome consists of a circular chromosome with a size of 5,617,746 bp and a GC content of 62.86%. No plasmids were detected in the genome. There were 5097 protein-coding sequences (CDSs) predicted in the genome. Phylogenetic analyses based on genomes of related *Pseudomonas* spp. identified strain JP233 as *Pseudomonas asiatica*. Comparative pangenomic analysis among 9 *P. asiatica* strains identified 4080 core gene clusters and 111 singleton genes present only in JP233. Genes associated with 2KGA production detected in strain JP233, included those encoding glucose dehydrogenase, pyrroloquinoline quinone and gluoconate dehydrogenase. Genes associated with mechanisms of plant-growth promotion and nutrient acquisition detected in JP233 included those involved in IAA biosynthesis, ethylene catabolism and siderophore production. Numerous genes associated with other properties beneficial to plant growth were also detected in JP233, included those involved in production of acetoin, 2,3-butanediol, trehalose, and resistance to heavy metals. This study provides the genetic basis to elucidate the plant-growth promoting and bio-remediation properties of strain JP233 and its potential applications in agriculture and industry.

## 1. Introduction

Phosphorus (P) is an essential macronutrient for plant growth and development. It is indispensable for the synthesis of large numbers of important organic compounds involved in photosynthesis, respiration, energy conversion and reproduction [1]. Although P is omnipresent in soil, P-resources available for plant absorption and utilization are relatively small [2]. In order to increase crop yields, phosphate fertilizers are often applied in large quantities. However, only a small proportion of the applied phosphate fertilizer is absorbed by plants [3] with a significant amount of P bound in soil as inorganic phosphate relatively unavailable to plants [4]. Long-term and large-scale application of phosphorus fertilizers inefficiently utilized by plants can result in economic burdens on farmers and an overall reduction in availability of high-quality P resources [5]. Underutilization of phosphate fertilizers can also result in soil acidification, eutrophication of water resources and associated environmental problems [6]. Moreover, P is a non-renewable resource and its availability for use in plant fertilizers is predicted to become more limited in the coming decades [7,8].

The discovery and application of P-solubilizing micro-organisms provides opportunities to address these issues. Microbial P-solubilization was first reported in the early 20th century, with observations that some bacterial strains isolated from soil were capable of liberating phosphate from bone meal and phosphate ore [9,10]. Subsequently, numerous highly efficient soil-borne phosphorus-solubilizing bacteria (PSB) have been isolated from a range of agricultural and natural environments including diverse rhizosphere soils [11] and karst rocky deserts [12]. Phosphate-solubilizing micro-organisms can convert insoluble P into soluble forms (primarily phosphate) that can be readily absorbed and utilized by plants [6]. Since most soil P that is unavailable to plants is present as insoluble inorganic forms, PSBs can increase the availability of phosphate and enhance plant growth [6,8].

Mechanisms of P-solubilization by PSB are primarily associated with secretion of organic anions, including but not limited to, acetate, lactate, malate, oxalate, succinate, gluconate, ketogluconate, citric acid and tartaric acid [6,13]. These organic anions act by desorbing inorganic P and complex organic P compounds from soil particles, either by direct exchange or chelation of cation-P complexes [6,14]. Release of organic anions is also associated with proton extrusion, resulting in soil acidification that increases solubility of inorganic P salts [6]. Organic anions are also effective in solubilizing Ca, Fe, Al, and Zn-phytate salts, thereby enhancing access to organic P compounds for mineralization by enzyme hydrolysis [6,13,14]. Enzymes produced by PSB with phosphate mineralizing and solubilizing functions include acid phosphatases (AP) and alkaline phosphatases (ALP), phytases and C-P lyases [6,14].

In our recent study, a PSB *Pseudomonas* sp. strain JP233 was isolated from soil and its P-solubilizing mechanism was identified by metabolomics and HPLC analyses [15]. The effects of JP233 on P content in soil leachates were also analyzed by microcosm experiments. Non-target metabolomic analysis identified 2-keto gluconic acid (2KGA) as the principal active metabolite responsible for P solubilization. Further, HPLC analysis revealed that 2KGA rapidly accumulated in vitro to 19.33 mg/mL within 48 h. Inoculation of JP233 into maize rhizosphere soils significantly decreased molybdate reactive phosphorus and total phosphorus contents in soil leachates. Inoculation with strain JP233 also significantly increased the foliar and total biomass of maize plants [15]. However, the genetic basis of phosphate solubilization and plant-growth promotion (PGP) by JP233 remained unclear. The aim of this study was to resolve the taxonomic identity of strain JP233 and elucidate its P-solubilizing and PGP mechanisms via genomic sequence analysis.

## 2. Materials and Methods

### 2.1. Bacterial Strains

The *Pseudomonas* sp. JP233 was isolated from the cucumber rhizosphere soil of vegetable greenhouse in Shandong Province, China [15]. Strain JP233 was identified as a highly effective PSB (Figure S1) and released 258 mg/L soluble phosphorus in NBRIP medium containing 5 g/L insoluble Ca_3_(PO_4_)_2_ within 48 h [15]. Strain JP233 was stored in glycerol in a −80 °C freezer before use.

### 2.2. Genomic DNA Preparation and Genome Sequencing

For DNA extraction, the stored JP233 culture was streaked onto LB plate and incubated in a growth chamber at 28 °C for 3 days. A single pure colony was inoculated into a sterile tube containing 5 mL LB broth, which was then grown in an orbital shaker (180 rpm) for 48 h at 28 °C. Strain JP233 was harvested and genomic DNA was extracted using a Wizard^®^ Genomic DNA Purification Kit (Promega, Madison, WI, USA), following the manufacturer’s instructions.

The JP233 genome was sequenced using a combination of PacBio RS II Single Molecule Real Time (SMRT) and Illumina NovaSeq 600 sequencing platforms by Majorbio Bio-pharm Technology Co., Ltd. (Shanghai, China). For Illumina sequencing, DNA samples were sheared into 400 bp–500 bp fragments and used to build the sequencing library. The prepared library was then used for paired-end Illumina sequencing using the PE150 strategy. For PacBio sequencing, a 10 kb insert library was prepared and sequenced on one SMRT cell using standard methods.

### 2.3. Genome Assembly, Gene Prediction and Annotation

The complete JP233 genome sequence was assembled using both the PacBio reads and Illumina reads. The raw sequence reads were trimmed and assembled into contigs using unicycler v0.4.8 [16]. Circularization of contigs was performed to generate the complete genome. Illumina reads were used for error correction of PacBio assembly results using Pilon [17].

Protein coding sequences (CDSs) were predicted by the NCBI prokaryotic genome annotation pipeline and Glimmer v3.02 [18], and tRNAs and rRNAs were predicted by tRNAscan-SE v2.0 [19] and Barrnap v0.8, respectively. Tandem repeats and interspersed repeats were predicted by Tandem Repeats Finder v4.07b [20] and Repeatmasker v4.0.7 [21], respectively. The predicted CDSs were annotated from NR (Non-Redundant Protein Database), Swiss-prot, Pfam, Clusters of Orthologous Groups of Proteins (COG), Gene Ontology (GO) and Kyoto Encyclopedia of Genes and Genomes (KEGG). The sequence alignment tools used included Basic Local Alignment Search Tool (BLAST) v2.3.0, Diamond v0.8.35 and HMMER v3.1b2 [22,23,24]. The circular map of the JP233 genome was created by Circos v0.69-6 [25]. Islander v1.2 was used to predict genomic islands (GIs) [26]. PHAge Search Tool (PHAST) was applied to predict prophages [27]. Minced v3 was used to detect CRISPR-Cas [28].

### 2.4. Genome-Based Identification

Based on the 16S rRNA gene sequence (GenBank ID: MW990045), strain JP233 showed a sequence identity greater than 99% to strains of *Pseudomonas* species, including *P. putida*, *P. plecoglossicida*, *P. moteilii*, *P. asiatica*, showing a sequence identity bigger than 99%, and was identified as *Pseudomonas* sp. [15]. The genome sequence of strain JP233 was submitted to GCM type strain genome database (https://gctype.wdcm.org/, accessed on 3 July 2022) for species identification pipeline analysis [29]. RAxML v8 was selected for phylogenetic analysis [30]. The genome sequences of type strains of closely related species were downloaded from the NCBI, including *P. putida* (GCF_000412675.1), *P. asiatica* (GCF_009932335.1), *P. plecoglossicida* (GCF_003391255.1), *P. monteilii* (GCF_003671975.1), *P. juntendi* (GCF_021560075.1), and *P. parafulva* (GCF_000425765.1). All-against-all average nucleotide identity (ANI) was computed using pyani 0.3.0 [31] with the method ANIm. Digital DNA–DNA hybridization (dDDH) analysis was conducted using the Genome-to-Genome Distance Calculator version 3.0 online service (https://ggdc.dsmz.de/, accessed on 17 July 2022) [32].

### 2.5. Pangenome Analysis

Complete and near complete genomes (scaffold number < 10) of JP233 and eight other *P. asiatica* strains, namely RYU5 (GCF_009932335.1), C1 (GCF_014656565.1), C3 (GCF_014792105.1), MY545 (GCF_005223305.1), MY601 (GCF_005223225.1), MY660 (GCF_005223255.1), MY680 (GCF_005223195.1), and MY756 (GCF_005222715.1), were downloaded from NCBI for pangenome analysis using Anvi’o v7.1 [33]. Primary functional annotation in Anvi’o was conducted using anvi-gen-contigs-database and anvi-run-ncbi-cogs commands. Subsequent pangenome gene clustering was carried out using blastp via the anvi-pan-genome command (--num-threads 6, --mcl-inflation 10, --minbit 0.5, --use-ncbi-blast). The ordering of the pangenome display was determined using Euclidean distances and Ward linkage settings.

## 3. Results

### 3.1. General Properties and Functional Analysis of Pseudomonas sp. JP233 Genome

**Figure 1:**
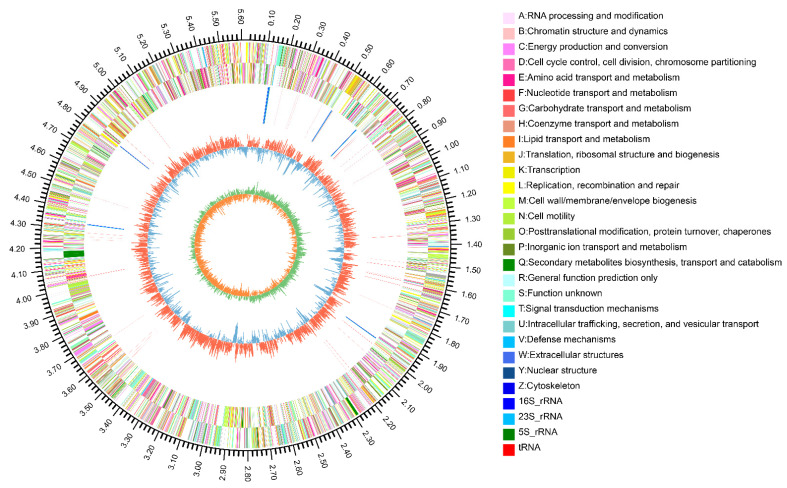
Circos plot of the *Pseudomonas* sp. JP233 genome. Circles from outer to inner respectively represent the genome size (Mbp), forward and reverses CDS indicating functional classifications, rRNA (blue radial lines) and tRNA (red radial lines), GC content (red and blue circles) and GC-skew (green and orange circles). Regarding GC content, red peaks indicate the greater GC richness in genomic regions relative to the genome average and blue peaks lower GC richness relative to the genome average. Genome GC-skew values (G − C/G + C) can assist in determining the leading and trailing lag chains. Generally, the leading chain′s GC-skew (green) is higher than 0, and the trailing lag chain′s GC-skew (orange) is lower than 0. GC-skew values can assist in determining the start and end points of replication in circular genomes.

The general properties of the JP233 genome are listed in Table 1. The genome consists of a circular chromosome with a size of 5,617,746 bp (Figure 1) and a GC content of 62.86%. No plasmids were detected in the sequenced DNA. The genome harbored 5039 protein coding genes (CDSs) and genes for 74 tRNAs and 22 rRNAs. JP233 was found to have nine CRISPRs, eight putative genomic islands, ranging from 6.9 kb to 61.1 kb in size, and three prophages ranging in size from 12.1 kb to 47.9 kb (Table 1 and Tables S1–S3). The whole genome sequence of strain JP233 was deposited in GenBank under the accession number CP107576.

The predicted 5039 genes of the JP233 genome were functionally analyzed using the COG, GO, and KEGG databases. According to the COG annotation, 4368 genes were classified into 21 specific categories (Supplementary Materials: Figure S2) and accounted for 85.70% of the total number of genes. The most frequently represented functional category was “amino acid transport and metabolism” (430 genes, 9.84%), followed by “transcription” (371 genes, 8.49%), “energy production and conversion” (280 genes, 6.41%), “inorganic ion transport and metabolism” (262 genes, 6.00%), and “cell wall/membrane/envelope biogenesis” (259 genes, 5.93%).

Based on GO annotation, 3809 genes were assigned to functions with 1782, 3126 and 1765 genes involved in cellular components, molecular functions, and biological processes, respectively (Supplementary Materials: Figure S3). The biological process “regulation of transcription, DNA-templated” (116 genes, 3.05%), cellular component “integral component of membrane” (1000 genes, 26.25%), and molecular function “DNA binding” (398 genes, 10.45%) were the most frequently represented gene categories.

According to KEGG pathway annotation, 2848 genes of JP233 were classified into 6 level-1 categories and 43 level-2 categories (Supplementary Materials: Figure S4). “Metabolism” (2310 genes, 81.14%) was the highest represented level-1 category. “Global and overview maps” (902 genes, 31.67%) was the most dominant level-2 category, followed by “amino acid metabolism” (313 genes, 10.99%), “carbohydrate metabolism” (250 genes, 8.78%), “membrane transport” (234 genes, 8.22%), and “signal transduction” (213 genes, 7.48%).

### 3.2. Phylogenomics of Pseudomonas sp. JP233

**Figure 2:**
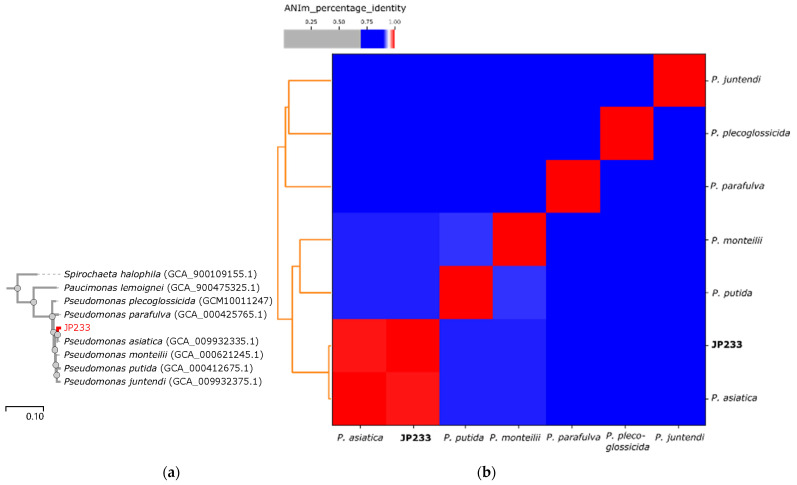
Identification of strain JP233 based on phylogenomic analyses. (**a**) Phylogenetic tree built with genomic sequences of JP233 and the type strains of related species by RAxML on gcType server (https://gctype.wdcm.org/, accessed on 3 July 2022). (**b**) Heatmap of ANIm percentage identities for JP233 and closely related *Pseudomonas* species. Cells in the heatmap corresponding to >95% ANIm sequence identity are shown in red. The adjoining dendrogram was constructed by linkage of ANIm percentage identities.

The gcType species identification pipeline analysis used 56 marker genes to perform phylogenetic analysis and showed that strain JP233 clustered with 6 *Pseudomonas* species, namely *P. plecoglossicida*, *P. parafulva*, *P. asiatica*, *P. monteilii*, *P. putida*, and *P. juntendi* (Figure 2a). *P. asiatica* was the closest relative to strain JP233 (Figure 2a). The reference genome sequences of the six type strains were then downloaded from NCBI and used for all-against-all ANI analysis with the JP233 genome. Strain JP233 and *P. asiatica* shared an ANI value greater than the 95% threshold value (Figure 2b). The ANI and dDDH values between *P. asiatica* type strain RYU5 and strain JP233 were 99.39% and 94.40% respectively, confirming the identity of strain JP233 as *P. asiatica* (Table S4).

### 3.3. Pangenomics of P. asiatica

**Figure 3:**
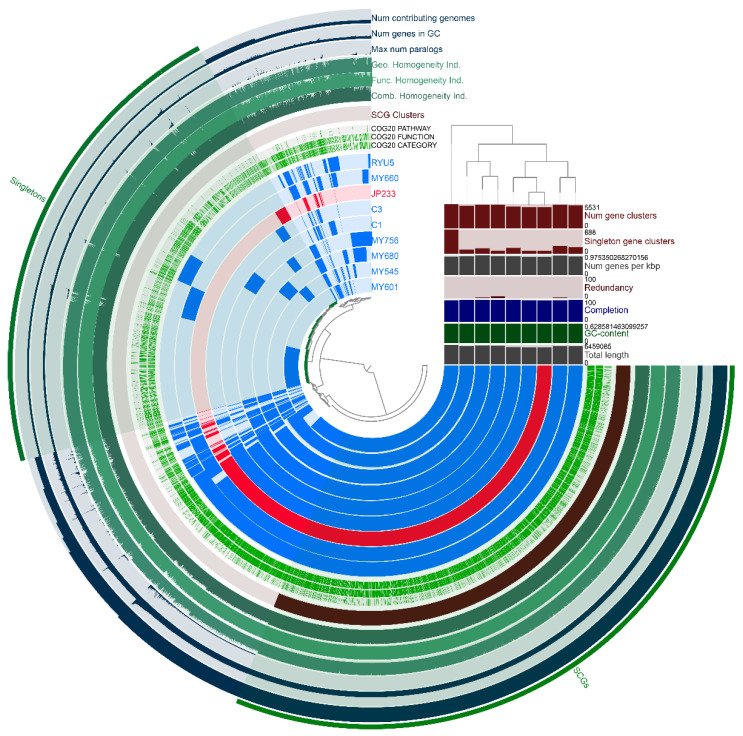
Pangenomic analysis of nine *P. asiatica* strains, generated by the Anvi’o. The ordering of the pangenome display was determined using Euclidean distances and Ward linkage settings. Beginning from the innermost ring and moving outward, rings 1 to 9 represent gene clusters identified in each of the *P. asiatica* genomes in the following order: 1, *P. asiatica* MY601; 2, *P. asiatica* MY545; 3, *P. asiatica* MY680; 4, *P. asiatica* MY756; 5, *P. asiatica* C1; 6, *P. asiatica* C3; 7, *P. asiatica* JP233; 8, *P. asiatica* MY660; and 9, *P. asiatica* RYU5. Rings 10 to 12 represents the location of known Clusters of Orthologous Genes (COG) categories, functions and pathways. Ring 13 highlights single-copy core gene clusters (SCGs). Rings 14 to 16 correspond to the combined, functional and geometric homogeneity index of each gene cluster. Rings 17 to 19 show the max number of paralogs, the number of genes within the identified gene clusters, and the number of contributing genomes. The histogram corresponds to number of gene clusters, number of singleton gene clusters, number of genes per kbp, genetic redundancy, completion, GC content, and total length for each *P. asiatica* genome present in the analysis. The dendrogram adjoining the histogram was constructed by gene cluster frequencies among the *P. asiatica* genomes.

**Figure 4:**
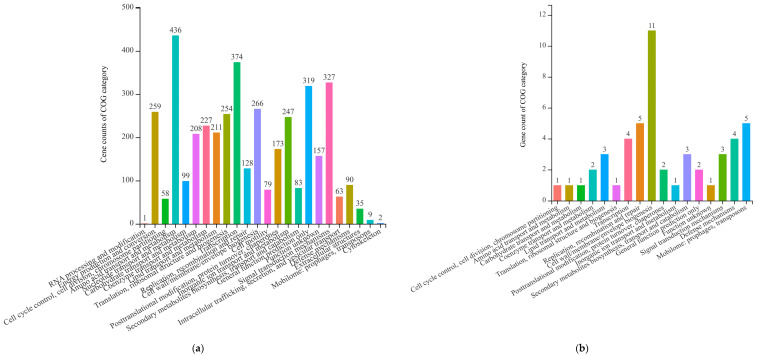
The Clusters of Orthologous Genes (COG) categories (**a**) among all 9 *P. asiatica* genomes and (**b**) singleton gene clusters of *P. asiatica* strain JP233.

The pangenomic analysis of 9 *P. asiatica* strains, including strain JP233, showed that the pangenome comprised 49,844 genes belonging to 8150 gene clusters (Figure 3). There were 4080 core gene clusters across all 9 genomes, illustrating the high genomic homogeneity within the species. Based on COG classification (Figure 4a), the most frequently represented functional categories among *P. asiatica* core gene clusters were “amino acid transport and metabolism” (436 genes, 10.69%), “transcription” (374 genes, 9.17%), “signal transduction mechanisms” (327 genes, 8.01%), “general function prediction only” (319 genes, 7.82%), and “cell wall/membrane/envelope biogenesis” (266 genes, 6.52%).

Singleton genes of the 9 *P. asiatica* strains comprised 2317 gene clusters. *P. asiatica* MY601 had the greatest number of singleton genes, with a total of 888 exclusive CDSs (16.05% of its total genome). There were 111 singleton genes identified in the JP233 genome among which only 47 genes (42.34% of them) could be assigned with COG annotations. The 47 COG-annotated genes were classified into 17 categories (Figure 4b), with the most frequently represented category being “cell wall/membrane/envelope biogenesis” (11 genes, 23.40%).

### 3.4. Plant Growth Promotion Related Genes in JP233

#### 3.4.1. Phosphate Solubilization

**Figure 5:**
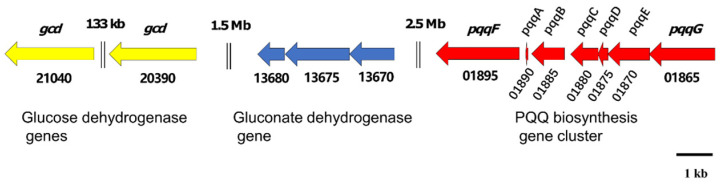
Schematic diagram of the genes associated with the synthesis of 2-keto gluconic acid in *P. asiatica* JP233 genome. jpw_21040 and 20390: glucose dehydrogenase genes (*gcd*); 13670–13680: gluconate dehydrogenase operon; 01865–01895: PQQ biosynthesis gene cluster. The genes are represented according to their size. Double vertical lines represent intervening DNA.

The direct oxidation of glucose to gluconic acid (GA) was proposed as the main metabolic steps for phosphate solubilization in pseudomonads [34]. The biosynthesis of GA is catalyzed by glucose dehydrogenase, which requires the cofactor pyrroloquinoline quinone (PQQ). Glucose is oxidized to GA in the periplasmic space, which can be further oxidized by membrane bound gluconate dehydrogenase to 2-keto gluconic acid (2KGA). Our previous metabolomics analysis indicated that 2KGA was the principal organic acid responsible for phosphate solubilization in JP233 [15]. Genes encoding glucose dehydrogenase (jpw_20390 and jpw_21040), the *pqqFABCDEG* operon (jpw_01865–jpw_01895) and the gluconate dehydrogenase operon (jpw_13670–jpw_13680) were detected in the JP233 genome (Figure 5).

The enzymes pyrophosphatase (PPA) and exopolyphosphatase (PPX) have also proposed to be involved in solubilization of inorganic phosphate by soil-borne microbes [35]. The enzyme PPX was recently reported to play an important role in transformation of inorganic polyphosphate to phosphate [36]. The *ppa* (jpw_02805) and *ppx* (jpw_24845) genes were both detected in strain JP233. Phosphatases and phytases are also known to release phosphate from recalcitrant organic P compounds [6,14]. Alkaline phosphatase produced by *P. asiatica* ZKB1 have been demonstrated to play a role in promoting plant growth [37]. Two alkaline phosphatase gene (jpw_06410 and jpw_08400) and a phytase-like protein gene (jpw_23245) were identified in strain JP233 (Table 2), but their efficacies to mineralize organic P and enhance phosphate availability to plants remain to be elucidated.

Two inorganic phosphate transport systems, phosphate inorganic transport (Pit) and phosphate specific transport (Pst), have been reported for the absorption and transport of inorganic phosphate from soil to bacteria [38]. Pit is a low-affinity, high-velocity phosphate absorption system that relies on proton motive force to generate ATP, whereas Pst is an ABC transporter with ATP-driven high-affinity phosphate uptake [39]. The Pst system is generally composed of PstS, PstC, PstA, PstB and PhoU [40]. The *pit* (jpw_20860) and *pstSCAB-phoU* operons (jpw_25360- jpw_25380) were identified in the JP233 genome. Notably, an additional copy of the *pstSCAB* operon (jpw_10985- jpw_11000) but lacking *phoU*, was also identified in the genome. The *pstSCAB-phoU* operon is controlled by the two-component regulatory system PhoBR, which regulates a large set of genes in response to low P concentrations [41]. The genes for PhoBR (jpw_25335 and jpw_25340) were also detected in *P. asiatica* JP233 (Table 2).

#### 3.4.2. Indole-3-Acetic Acid (IAA) Biosynthesis

**Figure 6:**
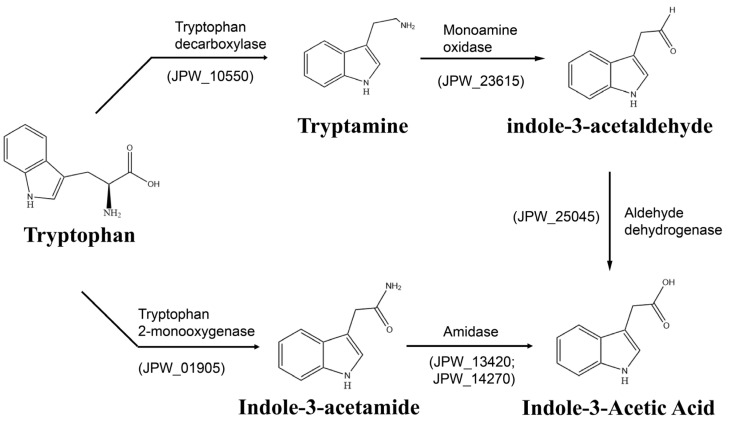
Proposed pathways for biosynthesis of IAA based on the annotation of *P. asiatica* JP233 genome.

IAA is the predominant form of auxin in plants and plays important roles in promoting cell division, elongation and differentiation during plant growth and development [42]. Many plant-associated microbes have the ability to synthesize IAA, and JP233 was shown to produce IAA in our previous metabolomics analysis [15]. At least two putative tryptophan-dependent IAA biosynthetic pathways were found in the JP233 genome, including the indole-3-acetamide (IAM) and the tryptamine (TAM) pathways (Figure 6). In the IAM pathway, tryptophan 2-mono-oxygenase (jpw_01905) is responsible for converting tryptophan to IAM, and amidase (jpw_13420 and jpw_14270) catalyzes the formation of IAA from IAM. In the TAM pathway, tryptophan is converted to TAM by the tryptophan decarboxylase (jpw_10550), and TAM is converted to indole-3-acetaldehyde (IAAld) by monoamine oxidase (jpw_23615). An aldehyde dehydrogenase (jpw_25045) then catalyzes the formation of IAA from IAAld. Regarding the secretion of IAA, three genes (jpw_04700, jpw_13635 and jpw_14640) encoding putative auxin efflux carriers were found in the JP233 genome.

#### 3.4.3. ACC Deaminase

Ethylene is another important plant hormone that affects plant growth, development and senescence [43]. High levels of ethylene can inhibit plant growth and, in more severe cases, cause plant death [44]. The substrate 1-aminocyclopropane-1-carboxylate (ACC) is present in root exudates and can be oxidized by ACC oxidase to produce ethylene. Some bacteria can promote plant growth by producing ACC deaminase, an enzyme that degrades ACC to α-ketobutyrate and NH_3_, thereby lowering the plant ethylene levels [45]. A gene (jpw_07635) coding for a pyridoxal-phosphate dependent enzyme was detected in the genome of JP233. Gene jpw_07635 showed high homology to ACC deaminase genes in the genomes of *Pseudomonas* sp. B22 (2017) (WP_085721703), *P. plecoglossicida* (WP_047595222) and *P. monteilii* (KXK71744), with amino acid sequence identities of 99.0%, 96.6% and 99.3%, respectively.

#### 3.4.4. Siderophores Production

**Figure 7:**
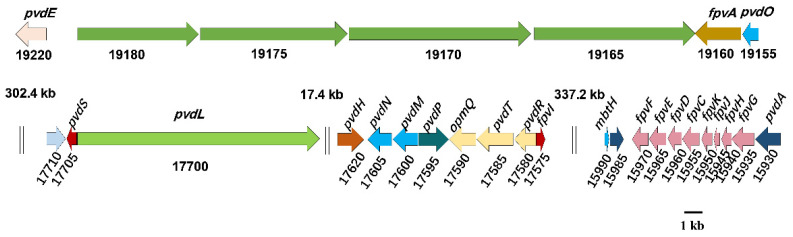
Schematic diagram of the gene clusters associated with the synthesis of pyoverdines (PVD) in *P. asiatica* JP233 genome. jpw_19220: *pvdE*; 19165–19180: NRPS genes; 19160: *fpvA*; 19155: *pvdO*; 17710: acetyltransferase gene; 17705: *pvdS*; 17700: *pvdL*; 17620: *pvdH*; 17595–17605: *pvdPMN*; 17590: *opmQ*; 17580–17585: *pvdRT*; 17575: *fpvI*; 15990: *mbtH*; 15985: thioesterase; 15955–15970: *fpvCDEF*; 15935–15950: *fpvGHJK*; 15930: *pvdA*. The genes are represented according to their size. Double vertical lines represent intervening DNA, the lengths of which are indicated in kb.

Siderophores are highly specific iron (Fe Ⅲ) chelators produced by microbes that play important roles for growth under iron-limiting conditions. Siderophores secreted by bacteria can competitively capture Fe to limit the growth of other microbes, including plant pathogens, thus participating in plant disease suppression [46]. Siderophores were also reported to promote solubilization of Fe-P complexes in soil [47]. Pyoverdines (PVDs) are yellow-green, fluorescent, high-affinity siderophores produced by pseudomonads, and are the most studied siderophores [48]. The genes associated with the synthesis of PVDs in the JP233 genome were mainly located in four gene clusters, separated by 302.4 kb, 17.4 kb and 337.2 kb, respectively (Figure 7). The proteins coded by these gene clusters have the following roles: (i) PvdL (jpw_17700) assembly of the peptide precursor of PVD and other non-ribosomal peptides synthetases (NRPSs) (jpw_19165–jpw_19180) responsible for adding specific amino acids to the peptide in the cytoplasm; (ii) PvdH (jpw_17620) and PvdA (jpw_15930) produces rare amino acids for the growing peptide; (iii) MbtH (jpw_15990) and the thioesterase (jpw_15985) are implied to act as auxiliary enzymes in PVD production; (iv) PvdE (jpw_19220) is an ABC transporter responsible for translocating the PVD precursor into the periplasm; (v) PvdO (jpw_19155) and PvdPMN (jpw_17595- jpw_17605) are involved in the PVD precursor maturation process; (vi) PvdRT and OpmQ (jpw_17580–jpw_17590) form a transport system to secret the mature PVD; (vii) FpvA (jpw_19160) is a ferripyoverdine receptor that binds iron-loaded PVD; and (viii) the *fpvGHJK* (jpw_15935–jpw_15950) and *fpvCDEF* (jpw_15955–jpw_15970) operons encode proteins constituting a system responsible for reducing Fe^3+^ to Fe^2+^, liberating PVD, and transporting Fe^2+^ to cytoplasm and (ix) PvdS (jpw_17705) and FpvI (jpw_17575) are sigma factors required for the regulation of PVD production and uptake (Table 2).

## 4. Discussion

*P. asiatica* was first described in 2019 [49], and has been isolated from water [50], soil [51], and human stool samples [49]. Strains of this species are functionally diverse and have been reported to be resistant to antimicrobials and heavy metals [50,52] and are capable of degrading the generally recalcitrant advanced glycation end products Nε-carboxymethyllysine and Nε–carboxyethyllysine [51]. *P. asiatica* C1 has been reported to aerobically synthesize coenzyme B_12_ and was developed as a microbial cell factory for the synthesis of 3-hydroxypropionic acid from glycerol [53]. *P. asiatica* ZKB1 has been shown to enhance availability of phosphate and promote plant growth, via P-solubilization and production of alkaline phosphatase [37]. In our recent study, *Pseudomonas* sp. strain JP233 was reported as a highly effective P-solubilizer capable of promoting maize growth and significantly reducing P leaching from soil [15]. In the present study, genomic analyses identified strain JP233 as *P. asiatica*. Currently (as of July 2022), there are 34 genome assemblies of *P. asiatica* strains publicly available on NCBI (https://www.ncbi.nlm.nih.gov/data-hub/genome/?taxon=2219225, accessed on 30 July 2022), with only 2 listed as complete. To our knowledge, this is the first report to describe comparative genomics of *P. asiatica* in detail.

The 34 *P. asiatica* genomes have a mean length of 6.01 Mb, mean GC content of 62.5% and a mean protein count of 5438. The genus *Pseudomonas* is one of the most complex Gram-negative genera and phylogenetic analyses based on sequences of the 16S rRNA, *rpoB*, *rpoD* and *gyrB* genes showed that species of *Pseudomonas* could be divided into 3 lineages and 13 groups, including the well-recognized *P. aeruginosa*, *P. fluorescens* and *P. putida* groups [54]. A recent study showed that *P. asiatica* is a member of the *P. putida* group and is closely related to *P. monteilii* and *P. putida* [49]. This result is consistent with the present study, based on comparative analysis of *P. asiatica*, *P. monteilii* and *P. putida* genome sequences. Whereas, the ANI and dDDH values between strain JP233 and the type strains of *P. fluorescens* and *P. aeruginosa* were only 84% and 22%, respectively (Table S4). The genome and PGP mechanisms of *P. monteilii* were rarely reported, so the comparison was mainly made between *P. asiatica* and *P. putida*.

Core genomic and pangenome analyses of 9 *P. putida* strains identified approximately 3386 genes [55]. In contrast, the present study identified 4080 core gene clusters for *P. asiatica*. However, actual core genome differences between these two species may be overestimated, due to the different analytical methods used. In the core genome of *P. putida*, the most abundant genes were those that encode transporters, enzymes and regulators for amino acid metabolism [55]. Similarly, in the present study the most frequently represented functional gene clusters in the core genome of *P. asiatica* were genes for amino acid transport and metabolism.

The production of organic acids has been shown to be associated with inorganic phosphate solubilization by many PSB [6,50]. Direct oxidation of glucose to gluconic acid (GA) was proposed to be the most important mechanism for this process in pseudomonads [34] and had been studied extensively [57]. The principal organic acid produced by strain JP233 during phosphate solubilization was 2KGA [15], synthesized via oxidation of GA by gluconate dehydrogenase. Genes responsible for 2KGA production from glucose, including glucose dehydrogenase (*gcd*), the enzyme cofactor *PQQ* (*pqq*) and gluconate dehydrogenase (*gad*), were all detected in the *P. asiatica* JP233 genome. The genes of the *pqq* operon show distinct differences in the number and genomic synteny among pseudomonads [58]. For example, whilst the gene order of *pqqABCDE* is conserved among *Pseudomonas* spp., *pqqF* and *pqqG* are found either proximal or distal to *pqqABCDE* [58]. The *pqqFABCDEG* arrangement in JP233 is identical to that of well-studied PSB *P. putida* KT2440, and the two strains share a high sequence similarity in their respective *pqq* operon and *gcd* genes [58]. Gluconate dehydrogenase is a key enzyme for the synthesis of 2KGA and generally contains flavoprotein, cytochrome c and γ subunits. In comparison to the industrial 2KGA producing strain *P. plecoglossicida* JUIM01, strain JP233 was identified to have amino acid similarities in the flavoprotein, cytochrome c and γ subunit of 81%, 79% and 61%, respectively [59]. 

Glucose catabolism in pseudomonads relies on the Entner–Doudoroff (ED) pathway, which utilizes 6-phosphogluconate (6-PG) as a key intermediate. 6-PG is formed via two pathways for the aforementioned oxidative synthesis of organic acids in the periplasm and the hexose phosphorylation in the cytoplasm. The oxidation pathway operates under aerobic conditions or high substrate availability, whereas the phosphorylation route predominates under oxygen- and/or glucose-limiting conditions. Glucose sequestration via its oxidation to acid derivatives, such as GA and 2KGA, was proposed to confer a competitive advantage to limit the use of this substrate by other micro-organisms [60,61].

Pyoverdine (PVD) siderophores are highly specific iron chelators that play important roles for growth of pseudomonads and also offer competitive advantages under iron-limiting conditions. Many distinct PVDs have been identified and comprise a conserved dihydroxyquinoline chromophore attached to peptide chains that exhibit inter- and intra- specific variation [62]. Both the chromophore and the peptide chain are synthesized by non-ribosomal peptide synthetases (NRPSs), which are highly variable due to the diversity of peptide moiety structures among PVDs [62]. Comparisons of PVDs gene clusters among *Pseudomonas* strains *P. aeruginosa* PAO1, *P. fluorescens* Pf0–1, *P. putida* KT2440, and *P. syringae* DC3000 revealed a suite of highly diverse NRPSs [48]. The PVD peptide precursor assembly gene PvdL and four NRPSs (jpw_19165–jpw_19180) required for synthesis of PVDs were detected in the genome of *P. asiatica* JP233. These NRPS genes ranged in size from 6.5 kb to 9.7 kb and were distinct from PVD-associated NRPS reported in these other pseudomonads. The genomic organization of PVD genes in strain JP233 was also different compared to these *Pseudomonas* spp. The PVD genes in *P. syringae* DC3000 form one large cluster, two clusters in *P. aeruginosa* PAO1, three in *P. fluorescens* Pf0–1 and *P. putida* KT2440 [48] and four gene clusters in *P. putida* LWPZE [63]. In *P. asiatica* JP233, the PVD genes are clustered into four regions separated by 302.4 kb, 17.4 kb, and 337.2 kb and are more dispersed than in *P. putida* LWPZE. In addition, the PVD gene order in strain JP233 is different to those reported in the above-mentioned pseudomonads [48].

Many bacteria have been reported to produce plant-growth regulators that can directly influence plant growth and development [38]. The auxin IAA is a plant hormone important for cell division, elongation and tissue differentiation and numerous plant-associated bacteria have been reported to synthesize IAA using tryptophan (Trp) as a precursor [35]. To date, five Trp-dependent IAA biosynthetic pathways have been identified in bacteria, including the indole-3-acetamide (IAM), indole-3-acetonitrile (IAN), indole-3-pyruvate (IPyA), tryptamine (TAM), and tryptophan side-chain oxidase (TSO) pathways [64]. Two of these IAA biosynthetic pathways, IAM and TAM, were identified in the genome of strain JP233. Both of these pathways have been previously reported in the *P. putida* BIRD-1 genome [65], whereas the IAM and IAN pathways were detected in *P. putida* strain LWPZF [63].

The enzyme ACC deaminase plays a key role in bacterial modulation of plant ethylene levels, which in turn, have been demonstrated to impact root development, flower senescence, and plant tolerance to biotic and abiotic stresses [66]. The gene coding for ACC deaminase, *acdS*, is reported to be prevalent in plant-associated bacteria, including *Pseudomonas* spp. [66]. Sequence alignments among *Pseudomonas acdSs* have identified conserved amino acid residues that are required for activity, including Lys51, Ser78, Tyr295, Glu296 and Leu322 [66]. The protein coded by the putative ACC deaminase gene of JP233 (jpw_07635) contain Lys51, Ser78 and Tyr295, but lacks Glu296 and Leu322. Similarly, the putative *acdS* in *P. putida* strain LWPZF also lacked Glu296 and Leu322, but was reported to show ACC deaminase activity [63]. Consequently, the putative *acdS* gene and ACC deaminase activity in strain JP233 require further investigation.

Bacteria, including *Pseudomonas* spp., have also been reported to produce acetoin and 2,3-butanediol, volatile compounds involved in regulating plant growth and abiotic stress tolerance [67,68]. Genome analysis of *P. asiatica* JP233 identified five genes related to the production of acetoin and 2,3-butanediol, including three acetolactate synthase large subunit genes (*ilvB*), the acetolactate synthase small subunit gene (*livH*) and the 2,3-butanediol dehydrogenase gene (*butB*). The sugar trehalose is an osmoprotectant also reported to confer tolerance to abiotic stresses, such as drought, high salinity and low temperature [69]. Trehalose biosynthetic pathways reported in bacteria include OtsA/OtsB, TreS, TreY/TreZ, TreP, and TreT [70]. In the present study, the *treS* (jpw_16785), *treY* (jpw_16755) and *treZ* (jpw_16745) genes of the TreS and TreY/TreZ pathways were detected in the genome of strain JP233.

More than 30 genes were identified in the *P. putida* core genome that serve as regulators and structural components of flagella [55]. We also identified 37 genes encoding flagellum-associated proteins within the *P. asiatica* core genome. Notably, three flagellar hook-related genes (*flgD*, *flgE* and *flgK*) and a flagellin-specific chaperone (*fliS*) were identified in the present study as having the lowest functional homogeneity among all single-copy *P. asiatica* core genes (Table S5). Flagella play a central role in adhesion, biofilm formation and chemotaxis of *Pseudomonas* spp., which enable bacteria to move toward more favorable conditions and to colonize diverse environments [56]. Consequently, the relatively low functional homogeneity of these genes may indicate diversification related to adaptability of *P. asiatica* to variable and dynamic environmental niches, including those encountered in soil and the plant rhizosphere.

Abiotic stresses associated with heavy metal contamination of soils, are also known to have significant deleterious impacts on plant growth [71]. Heavy metal tolerant bacteria have been used in bioremediation processes to improve plant growth in soils contaminated with heavy metals [72]. *P. asiatica* strains have previously been reported to be highly tolerant to heavy metals [50,52]. The present genome analysis of *P. asiatica* JP233 identified genes encoding tolerance to arsenic (*arsH*, *arsC*, *arsB*, *arsR*), copper (*cusR*, *cusS*, *copB*), chromium (*chrA* and *chrR*), zinc (*zntA*) and cadmium (*cadR*). Furthermore, two gene loci encoding the cobalt-zinc-cadmium efflux pump CzxCBA were also identified in strain JP233.

As discussed above, strain JP233 possesses an arsenal of mechanisms that are potentially related to plant growth-promotion activities. Although the functions of most genes have been identified in other pseudomonads, especially in the closely related *P. putida*, their functions need to be verified in strain JP233. This study provides the basis for future mechanistic studies of P-solubilization and plant-growth promotion by strain JP233. This could include differential gene expression and enzyme activity assays under P-limiting conditions, genetic manipulation of key target genes via site directed mutagenesis and complementation studies to confirm functional attributes in soil and the rhizosphere of host plants.

## 5. Conclusions

In the present study, we reported the genomic characteristics of the efficient phosphate-solubilizing and plant-growth promoting bacterium *Pseudomonas* sp. JP233. A phylogenetic genome-based analysis identified strain JP233 as *P. asiatica*. Comparative pangenomic analysis among 9 *P. asiatica* strains identified 4080 core gene clusters and 111 singleton genes present only in strain JP233. Genes coding for synthesis of 2-keto gluconic acid (2KGA), the principal metabolite responsible for phosphate solubilization by strain JP233, were detected in the bacterial genome. Other well-known mechanisms involved in plant-growth promotion and nutrient acquisition detected in JP233 included auxin (IAA) biosynthesis, ethylene catabolism and siderophore production. Numerous genes associated with abiotic stress tolerance and beneficial to plant growth were also detected, including production of acetoin, 2,3-butanediol, trehalose and resistance to heavy metals. Collectively, *P. asiatica* JP233 possesses an exceptional array of mechanisms to promote plant growth in challenging soil environments. This study provided the genetic basis to further investigate the plant-beneficial properties of strain JP233 and to explore potential inoculant applications in agriculture and industry.

## Figures and Tables

**Table 1:** Genome features of *Pseudomonas* sp. JP233.

Features	Total
Genome size (bp)	5,617,746
Chromosome	1
Plasmid	0
GC content (%)	62.86
Genes (total)	5139
CDSs (total)	5039
CDSs (with protein)	4968
tRNAs	74
rRNAs (5S, 16S, 23S)	22 (8, 7, 7)
ncRNAs	4
Pseudo Genes	71
CRISPR number	9
Genomic islands	8
Prophage	3
Interspersed repeats	44
Tandem repeats	80

**Table 2:** Genes related to plant growth promotion of JP233.

JP233 Gene ID	Gene	Product
Phosphate solubilization		
jpw_20390 jpw_21040	*gcd*	quinoprotein glucose dehydrogenase
jpw_01865	*pqqG*	S9 family peptidase
jpw_01870	*pqqE*	pyrroloquinoline quinone biosynthesis protein PqqE
jpw_01875	*pqqD*	pyrroloquinoline quinone biosynthesis protein PqqD
jpw_01880	*pqqC*	pyrroloquinoline-quinone synthase PqqC
jpw_01885	*pqqB*	pyrroloquinoline quinone biosynthesis protein PqqB
jpw_01890	*pqqA*	pyrroloquinoline quinone precursor peptide PqqA
jpw_01895	*pqqF*	pyrroloquinoline quinone biosynthesis protein PqqF
jpw_13670		gluconate 2-dehydrogenase cytochrome c subunit
jpw_13675		gluconate 2-dehydrogenaseαchain
jpw_13680		gluconate 2-dehydrogenaseγchain
jpw_20860	*pit*	inorganic phosphate transporter
jpw_10985 jpw_25380	*pstS*	phosphate ABC transporter substrate-binding protein PstS
jpw_10990 jpw_25375	*pstC*	phosphate ABC transporter permease subunit PstC
jpw_10995 jpw_25370	*pstA*	phosphate ABC transporter permease PstA
jpw_11000 jpw_25365	*pstB*	phosphate ABC transporter ATP-binding protein
jpw_25360	*phoU*	phosphate signaling complex protein PhoU
jpw_24845	*ppx*	
jpw_02805	*ppa*	
jpw_06410		alkaline phosphatase family protein
jpw_08400		alkaline phosphatase family protein
jpw_23245		esterase-like activity of phytase family protein
jpw_25335	*phoB*	two component winged helix family transcriptional regulator
jpw_25340	*phoR*	phosphate regulon sensor histidine kinase PhoR
IAA biosynthesis		
jpw_01905		tryptophan 2-mono-oxygenase
jpw_13420 jpw_14270	*amiE*	amidase
jpw_10550		tryptophan decarboxylase
jpw_23615		monoamine oxidase
jpw_25045		aldehyde dehydrogenase
jpw_04700 jpw_13635 jpw_14640		putative auxin efflux carriers
ACC deaminase activity		
jpw_07635		1-aminocyclopropane-1-carboxylate deaminase
Siderophore production		
jpw_17700	*pvdL*	non-ribosomal peptide synthetase
jpw_19165 jpw_19170 jpw_19175 jpw_19180		non-ribosomal peptide synthetase
jpw_17620	*pvdH*	aspartate aminotransferase family protein
jpw_15930	*pvdA*	lysine *N*(6)-hydroxylase/l-ornithine *N*(5)-oxygenase family protein
jpw_15990	*mbtH*	MbtH family protein
jpw_15985		thioesterase
jpw_19220	*pvdE*	cyclic peptide export ABC transporter
jpw_19155	*pvdO*	formylglycine-generating enzyme family protein
jpw_17595	*pvdP*	hypothetical protein
jpw_17600	*pvdM*	pyoverdine-tailoring dipeptidase-like protein PvdM
jpw_17605	*pvdN*	aminotransferase class V-fold PLP-dependent enzyme
jpw_17580	*macA*	efflux RND transporter periplasmic adaptor subunit
jpw_17585	*macB*	MacB family efflux pump subunit
jpw_17590	*opmQ*	efflux transporter outer membrane subunit
jpw_19160	*fpvA*	TonB-dependent siderophore receptor
jpw_15935	*fpvG*	PepSY domain-containing protein
jpw_15940	*fpvH*	hypothetical protein
jpw_15945	*fpvJ*	hypothetical protein
jpw_15950	*fpvK*	hypothetical protein
jpw_15955	*fpvC*	zinc ABC transporter substrate-binding protein
jpw_15960	*fpvD*	metal ABC transporter ATP-binding protein
jpw_15965	*fpvE*	metal ABC transporter permease
jpw_15970	*fpvF*	zinc ABC transporter substrate-binding protein
jpw_17705	*pvdS*	extracytoplasmic-function sigma-70 factor
jpw_17575	*fpvI*	sigma-70 family RNA polymerase sigma factor
jpw_17710		acetyltransferase
Trehalose		
jpw_16785	*treS*	trehalose synthase
jpw_16755	*treY*	malto-oligosyltrehalose synthase
jpw_16745	*treZ*	malto-oligosyltrehalose trehalohydrolase
Acetoin and 2,3-butanediol		
jpw_12445	*ilvB*	acetolactate synthase large subunit
jpw_19870	*ilvB*	acetolactate synthase large subunit
jpw_22100	*ilvH*	acetolactate synthase small subunit
jpw_22105	*ilvB*	acetolactate synthase 3 large subunit
jpw_02880	*butB*	2,3-butanediol dehydrogenase
Tolerance against metal toxicity		
jpw_11350	*arsH*	arsenical resistance protein ArsH
jpw_05755	*arsC*	arsenate reductase ArsC
jpw_03420	*arsB*	arsenic transporter
jpw_11360	*arsR*	metalloregulator ArsR/SmtB family transcription factor
jpw_08360 jpw_04220 jpw_20420	*cusR*	heavy metal response regulator transcription factor
jpw_08355 jpw_04215 jpw_20425	*cusS*	heavy metal sensor histidine kinase
jpw_02765	*copB*	copper resistance protein B
jpw_02775		copper resistance system multicopper oxidase
jpw_03360	*copZ*	heavy-metal-associated domain-containing protein
jpw_24395	*zntA*	heavy metal translocating P-type ATPase
jpw_24400	*cadR*	cadmium resistance transcriptional regulator CadR
jpw_10570	*chrA*	chromate efflux transporter
jpw_17220	*chrR*	class I chromate reductase ChrR
jpw_09995 jpw_09840	*czcA*	CusA/CzcA family heavy metal efflux RND transporter
jpw_09990 jpw_09835	*czcB*	efflux RND transporter periplasmic adaptor subunit
jpw_09985 jpw_09830	*czcC*	TolC family protein

## Data Availability

The data presented in this study are available on request from the corresponding author.

## Supplementary Materials

The following supporting information can be downloaded at: https://www.mdpi.com/article/10.3390/genes13122290/s1, Figure S1: Characteristic P solubilizing halo of PSB strain JP233 on an NBRIP plate, Figure S2: COG annotation of JP233, Figure S3: GO annotation of JP233, Figure S4: KEGG pathway annotation of JP233, Table S1: CRISPRs of JP233, Table S2: Genomic Islands of JP233, Table S3: Prophages of JP233, Table S4: ANI and dDDH values in comparisons of JP233 and type strains of the related *Pseudomonas* species, Table S5: Top 10 single-copy core gene clusters (SCG) with the least functional homogeneity among the 9 *P. asiatica* strains.

## References

[1] Shen J., Yuan L., Zhang J., Li H., Bai Z., Chen X., Zhang W., Zhang F. (2011). Phosphorus dynamics: From soil to plant. Plant Physiol..

[2] Zhu J., Li M., Whelan M. (2018). Phosphorus activators contribute to legacy phosphorus availability in agricultural soils: A review. Sci. Total. Environ..

[3] Zou X., Binkley D., Doxtader K.G. (1992). A new method for estimating gross phosphorus mineralization and immobilization rates in soils. Plant Soil.

[4] Hao X., Cho C.M., Racz G.J., Chang C. (2002). Chemical retardation of phosphate diffusion in an acid soil as affected by liming. Nutr. Cycl. Agroecosyst..

[5] Qiao J., Yang L., Yan T., Xue F., Zhao D. (2013). Rice dry matter and nitrogen accumulation, soil mineral N around root and N leaching, with increasing application rates of fertilizer. Eur. J. Agron..

[6] Harvey P.R., Warren R.A., Wakelin S. (2009). Potential to improve root access to phosphorus: The role of non-symbiotic microbial inoculants in the rhizosphere. Crop Pasture Sci..

[7] Cordell D., Drangert J.-O., White S. (2009). The story of phosphorus: Global food security and food for thought. Glob. Environ. Chang..

[8] Vitousek P.M., Porder S., Houlton B.Z., Chadwick O.A. (2010). Terrestrial phosphorus limitation: Mechanisms, implications, and nitrogen–phosphorus interactions. Ecol. Appl..

[9] Khan M.S., Zaidi A., Wani P.A., Lichtfouse E., Navarrete M., Debaeke P., Véronique S., Alberola C. (2009). Role of phosphate solubilizing microorganisms in sustainable agriculture—A review. Sustainable Agriculture.

[10] Sackett W., Patten A., Brown C. (1908). The solvent action of soil bacteria upon the insoluble phosphates of raw bone meal and natural raw rock phosphate. Cent. Bakteriol.

[11] Sperber J. (1958). Solution of apatite by soil microorganisms producing organic acids. Aust. J. Agric. Res..

[12] Xie J., Yan Z., Wang G., Xue W., Li C., Chen X., Chen D. (2021). A bacterium isolated from soil in a Karst rocky desertification region has efficient phosphate-solubilizing and plant growth-promoting ability. Front. Microbiol..

[13] Behera B.C., Singdevsachan S.K., Mishra R.R., Dutta S.K., Thatoi H.N. (2014). Diversity, mechanism and biotechnology of phosphate solubilising microorganism in mangrove—A review. Biocatal. Agric. Biotechnol..

[14] Kour D., Rana K.L., Kaur T., Yadav N., Yadav A.N., Kumar M., Kumar V., Dhaliwal H.S., Saxena A.K. (2021). Biodiversity, current developments and potential biotechnological applications of phosphorus-solubilizing and -mobilizing microbes: A review. Pedosphere.

[15] Yu H., Wu X., Zhang G., Zhou F., Harvey P.R., Wang L., Fan S., Xie X., Li F., Zhou H. (2022). Identification of the phosphorus-solubilizing bacteria strain JP233 and its effects on soil phosphorus leaching loss and crop growth. Front. Microbiol..

[16] Wick R.R., Judd L.M., Gorrie C.L., Holt K.E. (2017). Unicycler: Resolving bacterial genome assemblies from short and long sequencing reads. PLoS Comput. Biol..

[17] Walker B.J., Abeel T., Shea T., Priest M., Abouelliel A., Sakthikumar S., Cuomo C.A., Zeng Q., Wortman J., Young S.K. (2014). Pilon: An integrated tool for comprehensive microbial variant detection and genome assembly improvement. PLoS ONE.

[18] Delcher A.L., Bratke K.A., Powers E.C., Salzberg S.L. (2007). Identifying bacterial genes and endosymbiont DNA with Glimmer. Bioinformatics.

[19] Chan P.P., Lowe T.M., Kollmar M. (2019). tRNAscan-SE: Searching for tRNA genes in genomic sequences. Gene Prediction: Methods and Protocols.

[20] Benson G. (1999). Tandem repeats finder: A program to analyze DNA sequences. Nucleic Acids Res..

[21] Chen N. (2004). Using RepeatMasker to identify repetitive elements in genomic sequences. Curr. Protoc. Bioinform..

[22] Buchfink B., Reuter K., Drost H.-G. (2021). Sensitive protein alignments at tree-of-life scale using DIAMOND. Nat. Methods.

[23] Finn R.D., Clements J., Eddy S.R. (2011). HMMER web server: Interactive sequence similarity searching. Nucleic Acids Res..

[24] Johnson M., Zaretskaya I., Raytselis Y., Merezhuk Y., McGinnis S., Madden T.L. (2008). NCBIBLAST: A better web interface. Nucleic Acids Res..

[25] Krzywinski M., Schein J., Birol I., Connors J., Gascoyne R., Horsman D., Jones S.J., Marra M.A. (2009). Circos: An information aesthetic for comparative genomics. Genome Res..

[26] Bertelli C., Laird M.R., Williams K.P., Lau B.Y., Hoad G., Winsor G.L., Brinkman F.S., Simon Fraser University Research Computing Group (2017). IslandViewer 4: Expanded prediction of genomic islands for larger-scale datasets. Nucleic Acids Res..

[27] Zhou Y., Liang Y.J., Lynch K.H., Dennis J.J., Wishart D.S. (2011). PHAST: A fast phage search tool. Nucleic Acids Res..

[28] Bland C., Ramsey T.L., Sabree F., Lowe M., Brown K., Kyrpides N.C., Hugenholtz P. (2007). CRISPR Recognition Tool (CRT): A tool for automatic detection of clustered regularly interspaced palindromic repeats. BMC Bioinform..

[29] Wu L., Ma J. (2019). The Global Catalogue of Microorganisms (GCM) 10K type strain sequencing project: Providing services to taxonomists for standard genome sequencing and annotation. Int. J. Syst. Evol. Microbiol..

[30] Stamatakis A. (2014). RAxML version 8: A tool for phylogenetic analysis and post-analysis of large phylogenies. Bioinformatics.

[31] Pritchard L., Glover R.H., Humphris S., Elphinstone J.G., Toth I.K. (2016). Genomics and taxonomy in diagnostics for food security: Soft-rotting enterobacterial plant pathogens. Anal. Methods.

[32] Meier-Kolthoff J.P., Carbasse J.S., Peinado-Olarte R.L., Göker M. (2021). TYGS and LPSN: A database tandem for fast and reliable genome-based classification and nomenclature of prokaryotes. Nucleic Acids Res..

[33] Eren A.M., Esen Ö.C., Quince C., Vineis J.H., Morrison H.G., Sogin M.L., Delmont T.O. (2015). Anvi’o: An advanced analysis and visualization platform for ‘omics data. Peer J..

[34] Meyer J.B., Frapolli M., Keel C., Maurhofer M. (2011). Pyrroloquinoline quinone biosynthesis gene *pqqC*, a novel molecular marker for studying the phylogeny and diversity of phosphate-solubilizing Pseudomonads. Appl. Environ. Microbiol..

[35] Wu X., Rensing C., Han D., Xiao K., Dai Y., Tang Z., Liesack W., Peng J., Cui Z., Zhang F. (2022). Genome-resolved metagenomics reveals distinct phosphorus acquisition strategies between soil microbiomes. mSystems.

[36] Song H., Dharmasena M.N., Wang C., Shaw G.X., Cherry S., Tropea J.E., Jin D.J., Ji X. (2020). Structure and activity of PPX/GppA homologs from Escherichia coli and Helicobacter pylori. FEBS J..

[37] Bagewadi Z.K., Yaraguppi D.A., Mulla S.I., Deshpande S.H. (2022). Response surface methodology based optimization, partial purification and characterization of alkaline phosphatase isolated from Pseudomonas asiatica strain ZKB1 and its application in plant growth promotion. Mol. Biotechnol..

[38] Rosenberg H., Rosen B.P., Silver S. (1987). Phosphate transport in prokaryotes. Ion Transport in Prokaryotes.

[39] Santos-Beneit F., Rodríguez-García A., Franco-Domínguez E., Martín J.F. (2008). Phosphate-dependent regulation of the low- and high-affinity transport systems in the model actinomycete Streptomyces coelicolor. Microbiology.

[40] Sorger-Herrmann U., Taniguchi H., Wendisch V.F. (2015). Regulation of the pstSCAB operon in Corynebacterium glutamicum by the regulator of acetate metabolism RamB. BMC Microbiol..

[41] Lidbury I.D.E.A., Murphy A.R.J., Scanlan D.J., Bending G.D., Jones A.M.E., Moore J.D., Goodall A., Hammond J.P., Wellington E.M.H. (2016). Comparative genomic, proteomic and exoproteomic analyses of three Pseudomonas strains reveals novel insights into the phosphorus scavenging capabilities of soil bacteria. Environ. Microbiol..

[42] Ljung K. (2013). Auxin metabolism and homeostasis during plant development. Development.

[43] Iqbal N., Khan N.A., Ferrante A., Trivellini A., Francini A., Khan M.I.R. (2017). Ethylene role in plant growth, development and senescence: Interaction with other phytohormones. Front. Plant Sci..

[44] Glick B.R. (2014). Bacteria with ACC deaminase can promote plant growth and help to feed the world. Microbiol. Res..

[45] Glick B.R. (2012). Plant growth-promoting bacteria: Mechanisms and applications. Scientifica.

[46] Crichton R., Charloteauxwauters M. (1987). Iron transport and storage. Eur. J. Biochem..

[47] Zutter N.D., Ameye M., Vermeir P., Verwaeren J., Gelder L.D., Audenaert K. (2022). Innovative rhizosphere-based enrichment under P-limitation selects for bacterial isolates with high-performance P-solubilizing traits. Microbiol. Spectr..

[48] Ravel J., Cornelis P. (2003). Genomics of pyoverdine-mediated iron uptake in pseudomonads. Trends Microbiol..

[49] Tohya M., Watanabe S., Teramoto K., Uechi K., Tada T., Kuwahara-Arai K., Kinjo T., Maeda S., Nakasone I., Zaw N.N. (2019). Pseudomonas asiatica sp. nov., isolated from hospitalized patients in Japan and Myanmar. Int. J. Syst. Evol. Microbiol..

[50] Ramos M.S., Furlan J.P.R., Gallo I.F.L., dos Santos L.D.R., de Campos T.A., Savazzi E.A., Stehling E.G. (2020). High level of resistance to antimicrobials and heavy metals in multidrug-resistant Pseudomonas sp. isolated from water sources. Curr. Microbiol..

[51] Mehler J., Behringer K.I., Rollins R.E., Pisarz F., Klingl A., Henle T., Heermann R., Becker N.S., Hellwig M., Lassak J. (2022). Identification of Pseudomonas asiatica subsp. bavariensis str. JM1 as the first Nε-carboxy(m)ethyllysine-degrading soil bacterium. Environ. Microbiol..

[52] Tohya M., Uechi K., Tada T., Hishinuma T., Kinjo T., Ohshiro T., Maeda S., Kirikae T., Fujita J. (2021). Emergence of clinical isolates of Pseudomonas asiatica and Pseudomonas monteilii from Japan harbouring an acquired gene encoding a carbapenemase VIM-2. J. Med. Microbiol..

[53] Thi Nguyen T., Lama S., Kumar Ainala S., Sankaranarayanan M., Singh Chauhan A., Rae Kim J., Park S. (2021). Development of Pseudomonas asiatica as a host for the production of 3-hydroxypropionic acid from glycerol. Bioresour. Technol..

[54] Peix A., Ramírez-Bahena M.-H., Velázquez E. (2018). The current status on the taxonomy of Pseudomonas revisited: An update. Infect. Genet. Evol..

[55] Udaondo Z., Molina L., Segura A., Duque E., Ramos J.L. (2016). Analysis of the core genome and pangenome of Pseudomonas putida. Environ. Microbiol..

[56] Bouteiller M., Dupont C., Bourigault Y., Latour X., Barbey C., Konto-Ghiorghi Y., Merieau A. (2021). Pseudomonas flagella: Generalities and specificities. Int. J. Mol. Sci..

[57] Liang J., Liu J., Jia P., Yang T., Zeng Q., Zhang S., Liao B., Shu W., Li J. (2020). Novel phosphate-solubilizing bacteria enhance soil phosphorus cycling following ecological restoration of land degraded by mining. ISME J..

[58] An R., Moe L.A. (2016). Regulation of PQQ-dependent glucose dehydrogenase activity in the model rhizosphere dwelling bacterium Pseudomonas putida KT2440. Appl. Environ. Microbiol..

[59] Wang D.-M., Sun L., Sun W.-J., Cui F.-J., Gong J.-S., Zhang X.-M., Shi J.-S., Xu Z.-H. (2019). A membrane-bound gluconate dehydrogenase from 2-keto-d-gluconic acid industrial producing strain Pseudomonas plecoglossicida JUIM01: Purification, characterization, and gene identification. Appl. Biochem. Biotechnol..

[60] Latrach Tlemçani L., Corroler D., Barillier D., Mosrati R. (2008). Physiological states and energetic adaptation during growth of Pseudomonas putida mt-2 on glucose. Arch. Microbiol..

[61] Nikel P.I., Fuhrer T., Chavarría M., Sánchez-Pascuala A., Sauer U., de Lorenzo V. (2020). Redox stress reshapes carbon fluxes of Pseudomonas putida for cytosolic glucose oxidation and NADPH generation. bioRxiv..

[62] Ringel M.T., Brüser T. (2018). The biosynthesis of pyoverdines. Microb. Cell.

[63] Jin T., Ren J., Li Y., Bai B., Liu R., Wang Y. (2022). Plant growth-promoting effect and genomic analysis of the P. putida LWPZF isolated from C. japonicum rhizosphere. AMB Express.

[64] Patten C.L., Blakney A.J.C., Coulson T.J.D. (2013). Activity, distribution and function of indole-3-acetic acid biosynthetic pathways in bacteria. Crit. Rev. Microbiol..

[65] Roca A., Pizarro-Tobías P., Udaondo Z., Fernández M., Matilla M.A., Molina-Henares M.A., Molina L., Segura A., Duque E., Ramos J.L. (2013). Analysis of the plant growth-promoting properties encoded by the genome of the rhizobacterium P seudomonas putida BIRD-1. Environ. Microbiol..

[66] Glick B.R., Nascimento F.X. (2021). Pseudomonas 1-Aminocyclopropane-1-carboxylate (ACC) deaminase and its role in beneficial plant-microbe interactions. Microorganisms.

[67] Sharifi R., Ryu C.-M. (2018). Revisiting bacterial volatile-mediated plant growth promotion: Lessons from the past and objectives for the future. Ann. Bot..

[68] Ryu C.-M., Farag M.A., Hu C.-H., Reddy M.S., Wei H.-X., Paré P.W., Kloepper J.W. (2003). Bacterial volatiles promote growth in Arabidopsis. Proc. Natl. Acad. Sci. USA.

[69] Garg A.K., Kim J.-K., Owens T.G., Ranwala A.P., Choi Y.D., Kochian L.V., Wu R.J. (2002). Trehalose accumulation in rice plants confers high tolerance levels to different abiotic stresses. Proc. Natl. Acad. Sci. USA.

[70] Paul M., Primavesi L., Jhurreea D., Zhang Y. (2008). Trehalose metabolism and signaling. Annu. Rev. Plant. Biol..

[71] Tirry N., Tahri Joutey N., Sayel H., Kouchou A., Bahafid W., Asri M., El Ghachtouli N. (2018). Screening of plant growth promoting traits in heavy metals resistant bacteria: Prospects in phytoremediation. J. Genet. Eng. Biotechnol..

[72] Ahemad M. (2019). Remediation of metalliferous soils through the heavy metal resistant plant growth promoting bacteria: Paradigms and prospects. Arab. J. Chem..

