# Supplementary material for: Genomic Analysis of Pseudomonas asiatica JP233: An Efficient Phosphate-Solubilizing Bacterium
Source: Genes (Basel). 2022 Dec 5;13(12):2290. doi: 10.3390/genes13122290 (PMC9777792; doi:10.3390/genes13122290)
Supplement: Supplementary file 1 [file genes-13-02290-s001.zip › genes-2028583-supplementary.pdf]

Genomic analysis of *Pseudomonas asiatica* JP233, an efficient phosphate-solubilizing bacteria

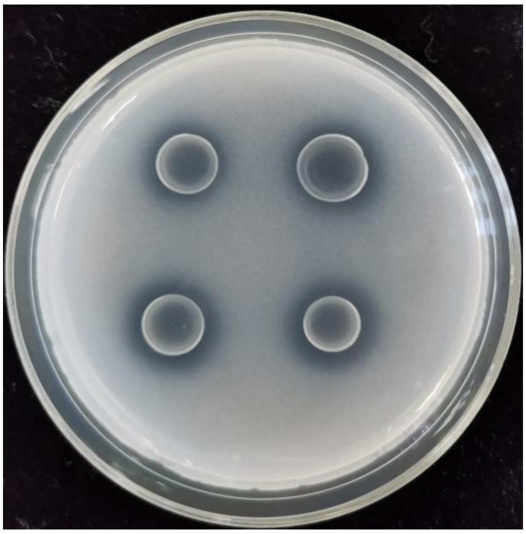

Figure S1. Characteristic P solubilizing halo of PSB strain JP233 on an NBRIP plate

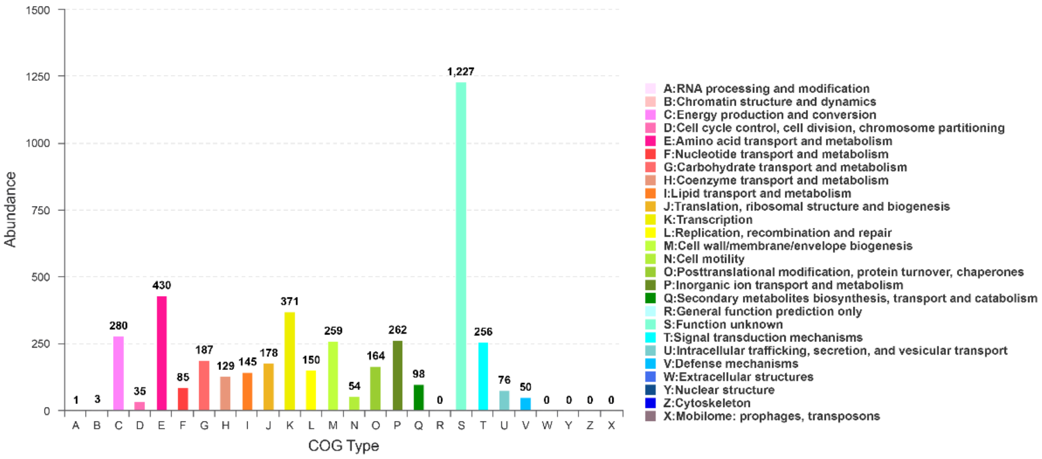

Figure S2. COG annotation of JP233.

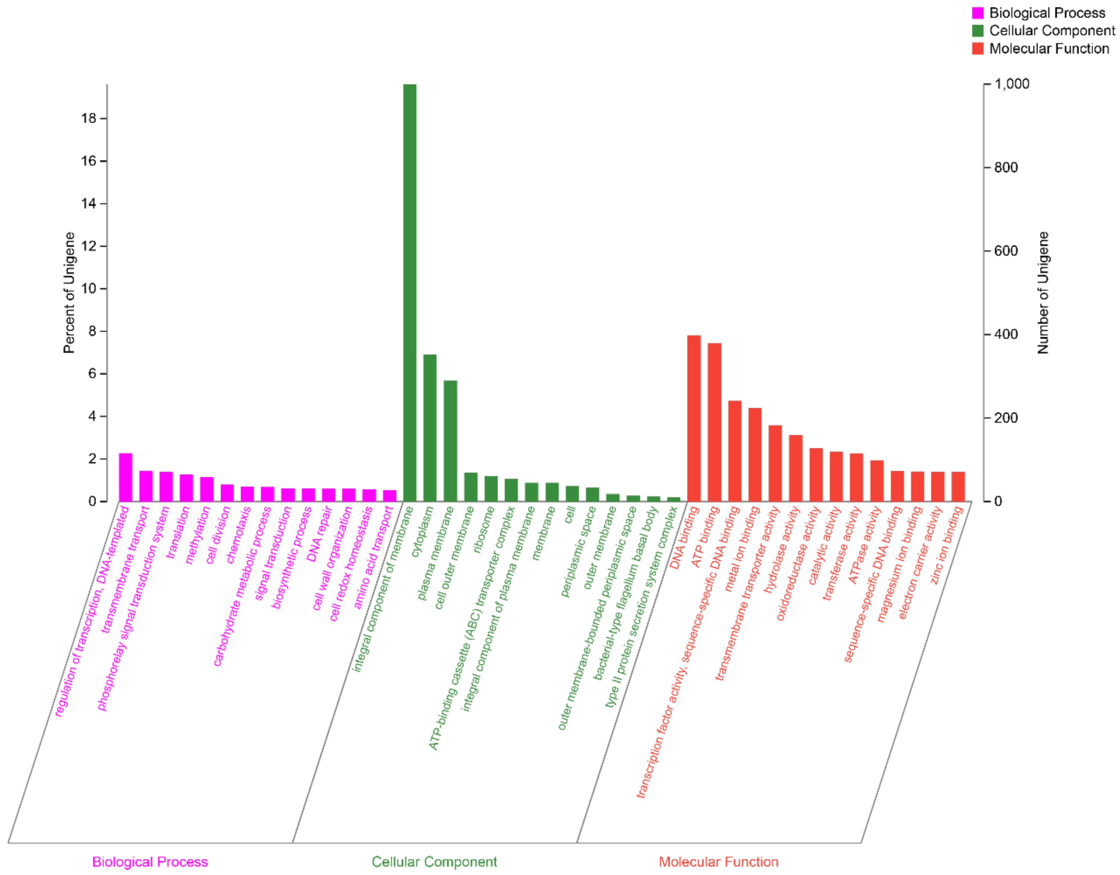

Figure S3. GO annotation of JP233.

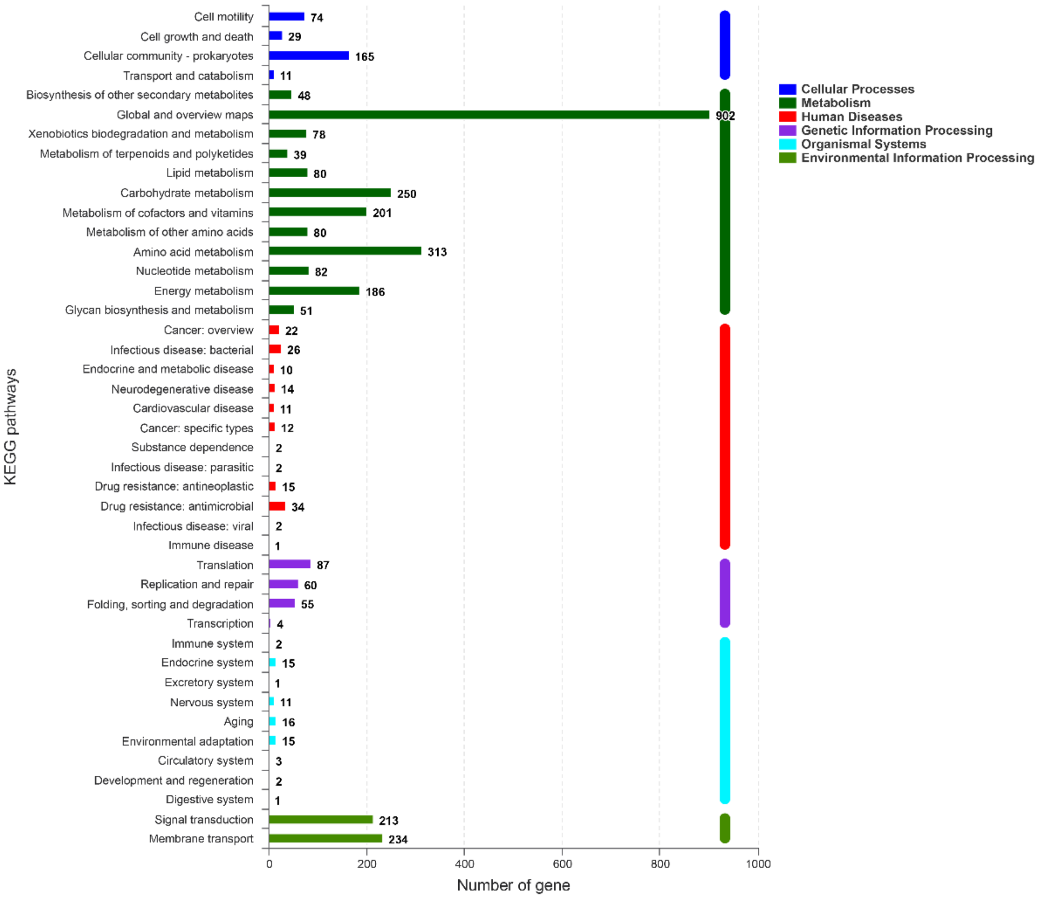

Figure S4. KEGG pathway annotation of JP233.

Table S1. CRISPRs of JP233.

CRISPR ID	Start	End	the number of repeats	average length of repeat (bp)	average length of spacers (bp)
CRISPR1	2269814	2269917	2	34	36
CRISPR2	2343288	2343394	2	36	35
CRISPR3	3160291	3160372	2	28	26
CRISPR4	3345495	3345590	2	34	28
CRISPR5	3843757	3843850	2	32	30
CRISPR6	3905289	3905440	3	23	41
CRISPR7	3966480	3966572	2	31	31
CRISPR8	4516575	4516826	4	27	48
CRISPR9	5045170	5045259	2	29	32

Table S2. Genomic Islands of JP233.

Island ID	Island Start	Island End	Length (kb)	CDS number
GI01	1450120	1471415	21.3	19
GI02	1863332	1878265	14.9	12
GI03	2520502	2549016	28.5	25
GI04	3387298	3448439	61.1	71
GI05	3560633	3567561	6.9	8
GI06	3870745	3887665	16.9	12

GI07	4261828	4274008	12.1	15
GI08	429492	455675	26.2	15

Table S3. Prophages of JP233.

Prophage ID	Ph Start	Ph End	Length (kb)	CDS Number	Possible Phage	GC content (%)
Ph01	1825633	1866138	40.5	50	Vibrio_phage_VHML_NC_004456	61.37
Ph02	3401946	3449855	47.9	102	Pseudomonas_phage_D3_NC_002484	60.01
Ph03	3712956	3725073	12.1	117	Pseudomonas_phage_phiCTX_NC_003278	63.1

Table S4. ANI and dDDH values in comparisons of JP233 and type strains of the related *Pseudomonas* species.

Query	Subject	ANI (%)	dDDH (%)
JP233	*P. asiatica* (GCF_009932335.1)	99.39	94.4
	P. putida (GCF_000412675.1)	90.51	40.2
	P. monteilii (GCF_003671975.1)	90.41	40.7
	P. juntendi (GCF_021560075.1)	89.04	35.7
	P. plecoglossicida (GCF_003391255.1)	88.50	33.3
	P. parafulva (GCF_000425765.1)	87.34	30.6
	P. fluorescens (NZ_LT907842.1)	84.37	22.1
	P. aeruginosa (NC_002516.2)	84.27	22.1

Table S5. Top 10 single-copy core gene clusters (SCG) with the least functional homogeneity among the 9 *P. asiatica* strains.

Gene cluster id	Functional homogeneity index	Geometric homogeneity index	COG20 FUNCTION ACC	COG20 FUNCTION
GC_00002844	0.799403697	0.991146875	COG1843	Flagellar hook-capping protein FlgD (FlgD) (PDB:6IEF)
GC_00004056	0.8122251	0.989432296	COG1256	Flagellar hook-associated protein FlgK (FlgK) (PDB:2D4Y)
GC_00002665	0.831590373	0.979473129	COG1749	Flagellar hook protein FlgE (FlgE) (PDB:3A69)
GC_00003038	0.87125448	0.971418445	COG1516	Flagellin-specific chaperone FliS (FliS) (PDB:1ORJ)
GC_00004037	0.873557157	0.936854401	COG1236	RNA processing exonuclease, beta-lactamase fold, Cft2 family (YSH1) (PDB:2DKF)
GC_00004174	0.878856438	0.913761829	COG1091	dTDP-4-dehydrorhamnose reductase (RfbD) (PDB:1KBZ)
GC_00004096	0.883053163	0.973137362	COG1086	NDP-sugar epimerase, includes UDP-GlcNAc-inverting 4,6-dehydratase FlaA1 and capsular polysaccharide biosynthesis protein EpsC (FlaA1) (PDB:2GN4)
GC_00002184	0.908331111	0.732209958	COG1914	Mn2+ or Fe2+ transporter, NRAMP family (MntH) (PDB:5KTE)

GC_00003753	0.908832627	0.983662603	COG0553	Superfamily II DNA or RNA helicase, SNF2 family (HepA) (PDB:6UXV)
GC_00001606	0.913764511	1	COG2200	EAL domain, c-di-GMP-specific phosphodiesterase class I (or its enzymatically inactive variant) (EAL) (PDB:2BAS)
